# Supplementary material for: Identification of Small Regions of Overlap from Copy Number Variable Regions in Patients with Hypospadias
Source: Int J Mol Sci. 2022 Apr 12;23(8):4246. doi: 10.3390/ijms23084246 (PMC9027593; doi:10.3390/ijms23084246)
Supplement: Supplementary file 1 [file ijms-23-04246-s001.zip › ijms-1613642-supplementary.pdf]

Supplementary Tables

Table S1 (Supplementary Table 1): Smallest Regions of Overlap (SROs)

For this study, genes within SROs with HI scores ≤ 10% or pLI scores ≥ 0.9 that have not been previously published in association with hypospadias are defined as candidate genes. Each candidate gene was investigated for any prior association with hypospadias by conducting a thorough literature review and query of OMIM® [1].

SRO ID	Chr.	SRO Type	Source(s)	# Del.	# Dup.	Total CNVs in SRO	# of CNVs from WUSTL	Coordinates (GRCh37/hg19)	Locus (GRCh37/hg19)	Size (kb)	Genes	Gene Content	# of genes with HI ≤10%	Genes with HI ≤10%	# of genes with pLI ≥ 0.9	Genes with pLI ≥ 0.9
SRO001	1	Simple (S)	DECIPHER (D)	3	2	5	-	chr1:1391996-1460043	1p36.33	68.05	3	*ATAD3A, ATAD3B, ATAD3C*	0	-	0	-
SRO002	1	Extrapolated (E)	D	3	1	4	-	chr1:145479218-145560475	1q21.1	81.26	6	*ANKRD35, GNRHR2, ITGA10, LIX1L, PEX11B, RBM8A*	0	-	1	***RBM8A****
SRO003	1	S	D + WUSTL (W)	3	2	5	1	chr1:145560476-145589918	1q21.1	29.44	3	*ANKRD35, NUDT17, PIAS3*	0	-	1	***PIAS3****
SRO004	1	E	D	3	1	4	-	chr1:145589918-145693383	1q21.1	103.47	2	*POLR3C, RNF115*	0	-	0	-
SRO005	1	S	D	1	5	6	-	chr1:146736136-147077663	1q21.1-21.2	341.53	6	*BCL9, CHD1L, LINC00624, OR13Z1P, OR13Z2P, OR13Z3P*	0	-	0	-
SRO006	1	S	D	3	0	3	-	chr1:187085032-187336720	1q31.1	251.69	1	*LINC01036*	0	-	0	-
SRO007	1	S	D + W	13	2	15	6	chr1:243760374-243769027	1q44	8.65	1	*AKT3*	1	***AKT3****	1	***AKT3****
SRO008	1	S	D + W	8	3	11	2	chr1:246369598-246384914	1q44	15.32	1	*SMYD3*	0	-	0	-
SRO009	1	S	D	6	3	9	-	chr1:248250872-248429279	1q44	178.41	6	*OR2L13, OR2M1P, OR2M2, OR2M3, OR2M4, OR2M5*	0	-	0	-
SRO010	2	S	D + W	3	2	5	1	chr2:242013323-242016876	2q37.3	3.55	2	*MTERFD2, SNED1*	0	-	1	***SNED1****
SRO011	4	E	D	5	1	6	-	chr4:188462419-188944050	4q35.2	481.63	2	*ADAM20P3, ZFP42*	0	-	0	-
SRO012	4	S	D + W	5	2	7	1	chr4:188944050-188953780	4q35.2	9.73	0	-	-	-	-	-

SRO013	4	E	D	5	1	6	-	chr4:188953780-189047790	4q35.2	94.01	2	RNU6-173P, TRIML2	0	-	0	-
SRO014	4	E	D + W	7	1	8	2	chr4:190195648-190199252	4q35.2	3.61	0	-	0	-	0	-
SRO015	4	E	D + W	6	1	7	1	chr4:190392962-190431430	4q35.2	38.47	0	-	0	-	0	-
SRO016	4	S	D + W	23	2	25	1	chr4:7212835-7361544	4p16.1	148.71	2	MIR4798	0	-	0	-
SRO017	4	S	D + W	23	2	25	1	chr4:7474595-7543439	4p16.1	68.85	1	SORCS2	0	-	0	-
SRO018	5	E	D	0	3	3	-	chr5:176667394-176904798	5q35.3	237.41	14	DBN1, F12, GRK6, LMAN2, MXD3, NSD1, PFN3, PRELID1, PRR7, PRR7-AS1, RAB24, RGS14, RN7SL562P, SLC34A1	0	-	2	**DBN1,* NSD1***
SRO019	6	S	D + W	5	3	8	1	chr6:168190220-168193993	6q27	3.77	1	C6orf123 (lncRNA)	0	-	0	-
SRO020	6	S	D	4	3	7	-	chr6:170676006-170911240	6q27	235.235	4	FAM120B, PDCD2, PSMB1, TBP	1	**TBP***	0	-
SRO021	7	S	D	3	1	4	-	chr7:101759560-101893297	7q22.1	133.74	1	CUX1	1	**CUX1***	1	**CUX1***
SRO022	7	S	D + W	13	3	16	1	chr7:153285501-153298175	7q36.2	12.67	0	-	0	-	0	-
SRO023	7	S	D + W	13	3	16	1	chr7:154076257-154098477	7q36.2	22.22	1	DPP6	0	-	0	-
SRO024	7	S	D	14	1	15	-	chr7:155596228-156069441	7q36.3	473.21	1	SHH	1	SHH	1	SHH
SRO025	7	S	D	3	0	3	-	chr7:26268514-26381495	7p15.2	112.98	1	SNX10	0	-	0	-
SRO026	8	S	D + W	4	3	7	2	chr8:15952011-16040743	8p22	88.73	1	MSR1	0	-	0	-
SRO027	9	E	D	3	0	3	-	chr9:138932676-139136889	9q34.3	204.21	4	NACC2, C9orf69, LHX3, QSOX2	0	-	0	-
SRO028	9	S	D	3	0	3	-	chr9:18344603-18681851	9p22.2-22.1	337.25	3	ADAMTSL1, MIR3152, RN7SKP258	1	**ADAMTSL1***	0	-
SRO029	9	S	D	11	0	11	-	chr9:5670381-5830137	9p24.1	159.76	2	ERMP1, KIAA1432	0	-	0	-
SRO030	9	S	D	10	1	11	-	chr9:8436578-8769692	9p24.1	333.12	1	PTPRD	1	**PTPRD***	1	**PTPRD***
SRO031	10	S	D	7	1	8	-	chr10:6677672-6833269	10p14	155.6	1	LINC00707	0	-	0	-
SRO032	11	S	D	7	4	11	-	chr11:120569545-120624320	11q23.3	54.78	1	GRIK4	0	-	0	-

SRO033	11	S	D	6	5	11	-	chr11:134207813-134586188	11q25	378.38	2	*B3GAT1, GLB1L2*	0	-	0	-
SRO034	11	S	D	6	5	11	-	chr11:134758036-134944770	11q25	186.74	0	-	0	-	0	-
SRO035	11	S	D	9	0	9	-	chr11:27723264-27727884	11p14.1	4.62	1	*BDNF*	1	***BDNF****	1	***BDNF****
SRO036	11	S	D	8	1	9	-	chr11:32450653-32826193	11p13	375.54	5	*CCDC73, EIF3M, HNRNPA3P9, WT1, WT1-AS*	2	***EIF3M,**** *WT1*	1	***EIF3M****
SRO037	11	S	D	8	1	9	-	chr11:34651737-34764237	11p13	112.5	1	*EHF*	0	-	1	***EHF****
SRO038	11	S	D	2	2	4	-	chr11:60001-383554	11p15.5	323.55	17	*ATHL1, B4GALNT4, BET1L, CICP23, IFITM1, IFITM2, IFITM3, IFITM5, LINC01001, NLRP6, ODF3, OR4F2P, PSMD13, RIC8A, RNU6-447P, SCGB1C1, SIRT3*	0	-	1	***PSMD13****
SRO039	12	S	D	2	5	7	-	chr12:132508290-133082859	12q24.33	574.57	8	*DDX51, EP400, EP400NL, FBRSL1, GALNT9, MUC8, NOC4L, SNORA49*	0	-	1	***EP400****
SRO040	13	S	D	11	0	11	-	chr13:110800326-110818101	13q34	17.78	1	*COL4A1*	0	-	1	***COL4A1****
SRO041	15	E	D	2	2	4	-	chr15:22770421-23260485	15q11.2	490.07	7	*CYFIP1, ELMO2P1, GOLGA8I, NIPA1, NIPA2, TUBGCP5, WHAMMP3*	0	-	2	***CYFIP1,* NIPA2****
SRO042	16	S	D	0	6	6	-	chr16:16194388-16282557	16p13.11	88.17	2	*ABCC1, ABCC6*	0	-	0	-
SRO043	16	S	D	0	3	3	-	chr16:28824778-29039612	16p11.2	214.84	11	*ATP2A1, ATXN2L, CD19, LAT, MIR4517, MIR4721, NFATC2IP, RABEP2, SH2B1, SPNS1, TUFM*	0	-	2	***ATXN2L,* SH2B1****
SRO044	16	E	D	1	2	3	-	chr16:29652360-29673954	16p11.2	21.6	1	*SPN*	0	-	0	-
SRO045	16	E	D	3	2	5	-	chr16:29673954-30190593	16p11.2	516.64	28	*ALDOA, ASPHD1, C16orf54, C16orf92, CDIPT, CDIPT-AS1, DOC2A, FAM57B, GDPD3, HIRIP3, INO80E, KCTD13, KIF22, MAPK3, MAZ, MVP, PAGR1, PPP4C, PRRT2, QPRT, RN7SKP127, SEZ6L2, SPN, TAOK2, TBX6, TMEM219, YPEL3, ZG16*	2	***CDIPT,* MAPK3****	2	***MAZ,* TAOK2****
SRO046	16	S	D	1	4	5	-	chr16:6842207-6963987	16p13.3	121.78	2	*RBFOX1, RNU6-457P*	1	***RBFOX1****	1	***RBFOX1****
SRO047	16	S	D	0	5	5	-	chr16:88554894-88653994	16q24.2	99.1	2	*ZC3H18, ZFPM1*	0	-	1	***ZC3H18****

SRO048	16	S	D	1	5	6	-	chr16:9826491-9864018	16p13.2	37.53	1	*GRIN2A*	0	-	1	***GRIN2A****
SRO049	17	S	D	2	3	5	-	chr17:1184539-1435764	17p13.3	251.23	7	*CRK, INPP5K, MYO1C, PITPNA, PITPNA-AS1, TUSC5, YWHAE*	3	***CRK,* PITPNA,* YWHAE****	2	***PITPNA,* YWHAE****
SRO050	17		D	2	1	3	-	chr17:59937184-60251560	17q23.2	314.38	5	*BRIP1, INTS2, MED13, RN7SL800P, POLRMTP1*	1	***MED13****	2	***INTS2,* MED13****
SRO051	17	S	D + W	10	3	13	9	chr17:7168108-7172631	17p13.1	4.52	0	-	0	-	0	-
SRO052	18	S	D	6	1	7	-	chr18:3332742-3762681	18p11.31	429.94	8	*DLGAP1, DLGAP1-AS1, DLGAP1-AS2, IGLJCOR18, RN7SL39P, RPL21P127, RPL31P59, TGIF1*	1	***DLGAP1****	1	***DLGAP1****
SRO053	22	S	D + W	1	12	13	5	chr22:16437771-16439957	22q11.1	2.19	0	-	0	-	0	-
SRO054	22	S	D	8	9	17	-	chr22:19217486-19295422	22q11.21	77.94	2	*CLTCL1, KRT18P62*	0	-	0	-
SRO055	22	S	D + W	8	10	18	1	chr22:21180259-21188442	22q11.21	8.18	1	*PI4KA*	0	-	0	-
SRO056	22	S	D + W	7	11	18	1	chr22:21282690-21312429	22q11.21	29.74	2	*CRKL, RN7SL389P*	1	***CRKL****	0	-
SRO057	22	S	D + W	10	8	18	1	chr22:22039876-22053776	22q11.21	13.9	2	*PPIL2, YPEL1*	0	-	0	-
SRO058	22	S	D + W	9	9	18	1	chr22:22297646-22332041	22q11.22	34.4	2	*PPM1F, TOP3B*	0	-	0	-
SRO059	22	S	D + W	8	20	28	13	chr22:23124497-23210365	22q11.22	85.87	9	*IGLV2-11, IGLV2-5, IGLV2-8, IGLV3-10, IGLV3-4, IGLV3-6, IGLV3-7, IGLV3-9, MIR650*	0	-	0	-
SRO060	22	S	D + W	8	8	16	1	chr22:23300886-23329428	22q11.22	28.54	0	-	0	-	0	-
SRO061	22	S	D + W	9	7	16	1	chr22:23337704-23346996	22q11.22	9.29	0	-	0	-	0	-
SRO062	22	S	D + W	9	7	16	1	chr22:23636086-23637830	22q11.23	1.74	1	*BCR*	1	***BCR****	1	***BCR****
SRO063	22	S	D	2	2	4	-	chr22:36323270-36406620	22q12.3	83.35	1	*RBFOX2*	1	*RBFOX2*	1	*RBFOX2*
SRO064	22	S	D	0	5	5	-	chr22:42067047-42569298	22q13.2	502.25	27	*C22orf46, CCDC134, CENPM, CYP2D6, CYP2D7P, CYP2D8P, FAM109B, HMGN2P10, LINC00634, MEI1, MIR33A, MIR378I, NAGA, NDUFA6, NDUFA6-AS1, NHP2L1, OLA1P1, RNU6-476P, RNU6ATAC22P, SEPT3, SHISA8, SLC25A5P1,*	0	-	2	***SREBF2,* TCF20****

												SMDT1, SREBF2, TCF20, TNFRSF13C, WBP2NL				
SRO065	22	S	D + W	0	6	6	1	chr22:42897269-42966416	22q13.2	69.15	6	*RN7SKP80, RNU6-513P, RRP7A, RRP7B, SERHL, SERHL2*	0	-	0	-
SRO066	22	E	D	2	6	8	-	chr22:50706851-50715515	22q13.33	8.67	2	*MAPK11, PLXNB2*	0	-	1	***PLXNB2****
SRO067	22	S	D + W	2	6	8	1	chr22:50953102-50956162	22q13.33	3.06	1	*NCAPH2*	0	-	0	-
SRO068	22	S	D + W	2	6	8	1	chr22:51093545-51095555	22q13.33	2.01	0	-	0	-	0	-
SRO069	22	S	D + W	1	5	6	3	chr22:51106829-51115526	22q13.33	8.77	1	*SHANK3*	0	-	1	***SHANK3****
SRO070	X	S	D + W	5	6	11	8	chrX:53475174-53477877	Xp11.22	2.7	0	-	0	-	0	-
SRO071	X	E	D + W	0	8	8	5	chrX:53477877-53478083	Xp11.22	0.21	0	-	0	-	0	-
SRO072	X	S	D + W	1	3	4	1	chrX:53480683-53488545	Xp11.22	7.86	0	-	0	-	0	-
SRO073	X	S	D + W	0	4	4	1	chrX:53587302-53588876	Xp11.22	1.58	1	*HUWE1*	0	-	1	***HUWE1****
SRO074	X	E	D	2	2	4	-	chrX:7964677-8115153	Xp22.31	150.48	1	*MIR651*	0	-	0	-
SRO075	X	E	D	2	1	3	-	chrX:8115153-8131752	Xp22.31	16.6	0	-	0	-	0	-
***Identified as candidate gene**																

Table S2 (Supplementary Table 2): Candidate Gene Tissue Specificity and Expression

For each of the 40 candidate genes, HPA was queried to determine specificity of tissue expression, as well as level of gene and protein expression in what HPA describes as “male tissues” (testis, seminal vesicles, epididymis, ductus deferens, and prostate) [2]. HPA records RNA expression sensitivity as either “enriched,” denoting RNA expression in a particular tissue, region, or cell type that is four times higher than any other tissue, region, or cell type, “group enriched,” “enhanced,” “low specificity,” or “not detected” [3]. Level of gene expression is recorded in HPA as Normalized Expression (NX) for each gene in various tissues and cell types, and protein-coding genes received a Protein Expression Score reported as “no data,” “not detected,” “low,” “medium,” or “high” [3]. *Note: No Protein Expression Scores were provided for ductus deferens.*

Gene	Tissue Expression Specificity	NX in Ductus Deferens	NX in Testis	Protein Expression Score in Testis	NX in Epididymis	Protein Expression Score in Epididymis	NX in Seminal Vesicle	Protein Expression Score in Seminal Vesicle	NX in Prostate	Protein Expression Score in Prostate

ADAMTSL1	Tissue enhanced (endometrium, retina)	6.5	2.3	Not detected	12	Not detected	5.6	Not detected	8.4	Not detected
AKT3	Low tissue specificity	17.4	10.4	Medium	12.3	Medium	23.9	Medium	19.4	Medium
ATXN2L	Low tissue specificity	22.8	77.2	Medium	22.9	High	34.2	Medium	29.2	Medium
BCR	Low tissue specificity	10.6	9.6	Medium	13.9	Medium	9.7	Medium	7	Medium
BDNF	Tissue enhanced (brain, smooth muscle)	0.7	1.3	Low	8.2	Not detected	4.2	Not detected	4.9	Not detected
CDIPT	Low tissue specificity	33.9	20.2	Medium	23	Low	38.8	Medium	36.8	Low
COL4A1	Tissue enhanced (placenta)	14.3	8.7	Low	21.8	Not detected	17.3	Not detected	24.6	Not detected
CRK	Low tissue specificity	31.5	17.9	Not detected	27.5	Not detected	27.3	Not detected	22.4	Low
CRKL	Low tissue specificity	12.3	12.6	Not detected	12.8	Medium	19.1	High	18.8	Low
CUX1	Low tissue specificity	17.3	8.3	Medium	17.4	Low	16.9	No data	22.8	Low
CYFIP1	Low tissue specificity	9.4	4.7	Medium	10.5	Medium	12	Medium	9.8	Medium
DBN1	Low tissue specificity	8.7	14.4	Medium	15	Medium	23	Not detected	14.9	Low
DLGAP1	Tissue enriched (brain)	4.1	4.3	No data	9	No data	11.4	No data	3.8	No data
EHF	Tissue enhanced (ductus deferens, salivary gland, seminal vesicle)	152.4	1.4	Not detected	21.4	Low	161.8	Not detected	20.7	Not detected
EIF3M	Low tissue specificity	26.5	43.5	High	38.9	Medium	30.3	Low	44.3	Medium
EP400	Low tissue specificity	13.4	27	High	25.8	High	22.6	High	15.6	Low
GRIN2A	Tissue enriched (brain)	0	5.2	Not detected	4.5	Not detected	1	Not detected	0.5	Not detected
HUWE1	Low tissue specificity	26.6	28.4	Medium	52.4	Medium	29.6	Medium	27.3	Low

INTS2	Low tissue specificity	5.3	12.9	Low	3.9	Not detected	3.2	Not detected	5.8	Not detected
MAPK3	Low tissue specificity	6.3	11.6	Medium	11.4	Medium	18.3	Medium	20.1	Medium
MAZ	Low tissue specificity	30.8	28.4	Medium	26.5	Low	31.2	Medium	48.1	Medium
MED13	Low tissue specificity	6.8	11.9	No data	9	No data	5.9	No data	7.1	No data
NIPA2	Low tissue specificity	23.9	19.5	Medium	18.3	Medium	14	Medium	20.6	Medium
NSD1	Low tissue specificity	17	28.1	High	13.9	Medium	18.7	High	14.2	No data
PIAS3	Low tissue specificity	16.5	10	Not detected	15	Not detected	26.8	Not detected	20.3	Not detected
PITPNA	Low tissue specificity	35	25	Not detected	29.8	High	33.2	Medium	27.6	Not detected
PLXNB2	Low tissue specificity	39.8	19.3	Medium	28	High	31	Medium	27.7	Medium
PSMD13	Low tissue specificity	20	21.2	Medium	20.3	Medium	21.1	Medium	24	Medium
PTPRD	Tissue enhanced (brain, parathyroid gland)	0.2	2.5	High	1.8	Not detected	2	Medium	3.3	Not detected
RBFOX1	Group enriched (brain, heart muscle, skeletal muscle)	0	1.7	Not detected	5	Not detected	4.9	Not detected	2.6	Not detected
RBM8A	Tissue enriched (blood)	9.8	8.5	High	5	High	6.3	Medium	5.7	High
SH2B1	Low tissue specificity	32.7	19.9	No data	21.6	No data	32.6	No data	25.4	No data
SHANK3	Tissue enhanced (brain)	0.1	3.4	Not detected	3.4	Medium	3.4	Not detected	1.9	Not detected
SNED1	Low tissue specificity	19.1	11.9	Medium	23.9	High	11.1	High	12.9	High
SREBF2	Low tissue specificity	22.3	13.5	High	41.5	Medium	20	Not detected	22.8	Not detected
TAOK2	Low tissue specificity	11.8	21.2	Low	13	Not detected	15.9	Low	13.6	Not detected

TBP	Low tissue specificity	15.2	51.4	Low	12.1	Low	15.5	Low	13.1	Low
TCF20	Low tissue specificity	4.4	9.3	Medium	4.3	Medium	4.2	Medium	4.4	Low
YWHAE	Low tissue specificity	23.8	46	High	54.1	High	27.7	High	35	Medium
ZC3H18	Low tissue specificity	17.4	31.4	High	12.2	High	13	High	13.2	High

Table S3 (Supplementary Table 3): Genes Previously Associated with Hypospadias

All genes published before April 2020 as being causative, risk factors, or candidate genes for hypospadias were identified. PubMed was searched to identify manuscripts describing the genetic etiology of hypospadias. We searched for papers in English published between January 1995 and April 2020 using the following keywords in the abstract or title: '(hypospadia OR hypospadias) AND (genetic OR genomic OR gene OR copy number) NOT surgery NOT surgical NOT repair NOT reconstruction NOT procedure NOT incised'. This search provided 613 results, of which the titles and abstracts were used to identify relevant papers. In addition, HPA [4] and OMIM® [1] were queried to identify additional associated genes. Specifically, the OMIM® query returned all genes and loci with hypospadias reported as a phenotype in at least one individual with a corresponding variant.

**Gene**	**Suspected Pathogenic Sequence Variation**	**Single-Gene Sequence Variation Associated with Isolated Hypospadias in Screening Studies**	**Identified as Risk Factors in Association Studies**	**Identified as Candidates in Gene Expression Studies**	**Proposed as Candidates given Role in Gonadal Development**
ADAT3 [5]	✓		✓		
AR [6–8]	✓	✓	✓		
ARNT2 [9]			✓		
ATF3 [7,8]		✓	✓		
BMP4 [7,8]		✓			
BMP7 [7,8,10]		✓	✓		
BNC2 [7,8]		✓			
CTGF [8]				✓	
CXorf6 [11]	✓				
CYP1A1 [6,8,12]			✓		
CYP1A2 [9]			✓		
CYP3A4 [6]		✓	✓		
CYP11A1 [13–15]	✓				
CYP17A1 [6]	✓		✓		

CYR61 [8]				✔	
DGKK [2,6,8]	✔		✔		
DMBT-1 [6]			✔		
DMRT1 [16]					✔
EFNB2 [17–19]	✔				
EGF [8]				✔	
EMX2 [20]			✔		
ESR1 [7,8]			✔		
ESR2 [7,8]			✔		
EYA1 [21]					
FGF8 [7,8]		✔	✔		
FGF10 [7,10]			✔		
FGFR2 [7,8]		✔	✔		
FKBP4 [7]					✔
FLNA [20]			✔		
FOG2 [8]			✔		
GATA4 [8]			✔		
GLI1 [10]			✔		
GLI2 [10]			✔		
GLI3 [10,16,22]		✔	✔		
GRID1 [23]					✔
GSTM1 [8]			✔		
GSTT1 [8]			✔		
HAAO [24]			✔		
HOXA4 [7,8]		✔			
HOXA13 [7,21]					✔
HOXB6 [7,8]		✔			
HOXD13 [8]			✔		
HSD3B1 [25]		✔	✔		
HSD3B2 [6–8]		✔	✔		
HSD17B3 [6–8,25]		✔	✔		
INSL3 [7]			✔		
IRX5 [7,24]					✔
IRX6 [21]			✔		
KIAA2022 [23]					✔

LHCGR [26]	✔				
MAMLD1 [2,6–8]	✔	✔	✔		
MID1 [2,7,8]	✔	✔			
MSX1 [8]			✔		
MYRF [27]	✔				
NR5A1 [7,8]		✔			
PPARGC1B [23]					✔
PPP1R12A [28]				✔	
PRSS3P2 [6]			✔		
PROKR2 [22]		✔			
PTCH1 [8]			✔		
RBFOX2 [29]				✔	
RET [6]			✔		
SHH [10,16]			✔		
SOX9 [7,16]					✔
SR4 [30]					✔
SRD5A1 [7]	✔		✔		
SRD5A2 [6–8]	✔	✔	✔		
SRY [7]					✔
STARD3 [6,25]		✔	✔		
STS [6,25]		✔	✔		
TGFBR2 [31]			✔		
TRIM17 [22]		✔			
TSC1 [6]			✔		
VAMP7 [32]					✔
WDR11 [20]			✔		
WNT5A [33]					✔
WT1 [7,8,10]	✔	✔	✔		
ZEB2 [8]	✔				
ZFHX3 [21]					✔
ZFPM2 [16,22]		✔			

Table S4 (Supplementary Table 4): Common Comorbidities

For SROs with at least 3 cases with a mixture of neurodevelopmental abnormalities as defined by Human Phenotype Ontology (e.g. intellectual disability, global developmental delay, delayed speech and language development) [34], the common phenotype was recorded as “neurodevelopmental abnormality.” The breakdown

of specific phenotypes was also recorded. If all cases within the SRO had a more specific phenotype (e.g. if all cases had global developmental delay), then the specific phenotype was listed.

SRO ID	Most Common Phenotype	Proportion of CNVs	2nd Most Common Phenotype	Proportion of CNVs	3rd Most Common Phenotype	Proportion of CNVs	4th Most Common Phenotype	Proportion of CNVs	5th Most Common Phenotype(s)	Proportion of CNVs
SRO003	Neurodevelopmental abnormality	3/5								
SRO005	Neurodevelopmental abnormality	6/6								
SRO007	Neurodevelopmental abnormality	9/15								
SRO008	Intellectual disability	9/11	Ventricular septal defect	6/11						
SRO010	Neurodevelopmental abnormality	5/5	Hypotonia	3/5						
SRO011	Intellectual disability	4/6	Feeding difficulties in infancy	3/6	Inguinal hernia	3/6				
SRO013	Intellectual disability	4/6	Feeding difficulties in infancy	3/6	Inguinal hernia	3/6				
SRO016	Intellectual disability	14/25								
SRO017	Hypertelorism	18/25	Low-set ears	11/25	Non-midline cleft lip	8/25	Talipes equinovarus	6/25	Hydrocephalus	3/24
SRO018	Delayed speech and language development	3/3								
SRO019	Hypoplastic nail	4/8	Abnormality of the pinna	3/8						
SRO021	Neurodevelopmental abnormality	3/4								
SRO023	Neurodevelopmental abnormality	10/16	Congenital heart defect	3/16						
SRO024	Intellectual disability	6/15								
SRO026	Intellectual disability	5/7								
SRO027	Intellectual disability	3/3								

SRO029	Cryptorchidism	7/11	Short stature	4/11	Small for gestational age	3/11	Hydronephrosis	3/11		
SRO030	Intellectual disability	7/11	Anteverted nares	6/11	Patent ductus arteriosus	4/11	Microcephaly	4/11		
SRO032	Anteverted nares	5/11	Microcephaly	3/11						
SRO034	Abnormality of the pinna	6/11	Short stature	3/11						
SRO036	Micrognathia	3/9								
SRO037	Short stature	7/9								
SRO040	Micrognathia	5/11	Hypertelorism	5/11	Anal atresia	4/11	Depressed nasal bridge	4/11	Cleft palate, blepharophimosis, low-set ears	3/11
SRO041	Delayed speech and language development	3/4								
SRO042	Intellectual disability	5/6	Long philtrum	4/6						
SRO047	Micropenis	4/5								
SRO048	Neurodevelopmental abnormality	5/6								
SRO049	Short neck	3/5								
SRO051	Neurodevelopmental abnormality	5/13	Low-set ears	3/13						
SRO052	Neurodevelopmental abnormality	7/7								
SRO053	Microcephaly	3/13	Micropenis	3/13						
SRO054	Preauricular skin tag	3/17								
SRO055	Neurodevelopmental abnormality	11/18	Hearing impairment	5/18						
SRO057	Cleft palate	6/18								
SRO067	Small for gestational age	3/8	Short stature	3/8						
SRO068	Intellectual disability	3/8								
SRO070	Neurodevelopmental abnormality	3/11								

SRO071	Neurodevelopmental abnormality	4/8								

Supplementary Table 5: Selected Associated Syndromes

SRO	Location	Size (kb)	SRO CNV Composition	Candidate Genes	Syndrome	Notes
SRO002	1q21.1	81.26	Mixed	*RBM8A*	Thrombocytopenia-Absent Radius Syndrome	-Syndrome known to be caused by CNVs and SNPs in *RBM8A* [1]<br>-These findings support a recent call to expand syndrome phenotype to include hypospadias [35]
SRO005	1q21.1-21.2	341.53	Mixed	None	Chromosome 1q21.1 Deletion and Duplication Syndromes	-Both syndromes associated with intellectual disability [1]; all of our cases had neurodevelopmental abnormalities
SRO018	5q35.3	237.41	All duplications	*DBN1, NSD1*	Sotos Syndrome	-Syndrome known to be due to mutations in *NSD1* [1]<br>-Syndrome associated with intellectual disability [1]; all SRO cases had delayed speech and language development
SRO041	15q11.2	490.07	Mixed	*CYFIP1, NIPA2*	Burnside-Butler Syndrome	-*CYFIP1* and *NIPA2* (both candidate genes) implicated in the syndrome [36]<br>-Syndrome associated with various developmental and psychiatric disorders [1]; 3 of 4 cases in SRO had delayed speech and language development
SRO042	16p13.11	88.17	All duplications	None	Chromosome 16p13.11 Deletion and Duplication Syndromes	-Both syndromes identified as conferring susceptibility to neurocognitive disorders [1]; 5 of 6 SRO cases had intellectual disability
SRO043	16p11.2	214.84	All duplications	*ATXN2L, SH2B1*	Chromosome 16p11.2 Deletion and Duplication Syndromes	-Both syndromes associated with susceptibility to Autism Spectrum Disorder [1]
SRO049	17p13.3	251.23	Mixed	*CRK, YWHAE, PITPNA*	Miller-Dieker Syndrome	-OMIM describes 1 case with hypospadias but does not list it as syndrome phenotype [1]
SRO062	22q11.23	1.74	Mixed	*BCR*	Chromosome 22q11.2 Distal Deletion Syndrome	
SRO068	22q13.33	2.01	Mixed	None	Chromosome 22q13 Deletion Syndrome (Phelan-McDermid Syndrome)	-Syndrome associated with intellectual disability [1]; 3 of 8 SRO cases had intellectual disability
SRO069	22q13.33	8.77	Mixed	*SHANK3*	Chromosome 22q13 Deletion Syndrome (Phelan-McDermid Syndrome)	
SRO070	Xp11.22	2.7	Mixed	None	Chromosome Xp11.22 Duplication Syndrome	-Syndrome strongly associated with intellectual disability [1]; 3 of 11 SRO cases had neurodevelopmental abnormality
SRO071	Xp11.22	0.21	All duplications	None	Chromosome Xp11.22 Duplication Syndrome	-Syndrome strongly associated with intellectual disability [1]; 4 of 8 SRO cases had neurodevelopmental abnormality
SRO072	Xp11.22	7.86	Mixed	None	Chromosome Xp11.22 Duplication Syndrome	

SRO073	Xp11.22	1.58	All duplications	*HUWE1*	Chromosome Xp11.22 Duplication Syndrome	

Supplementary Table References

1. McKusick-Nathans Institute of Genetic Medicine *Online Mendelian Inheritance in Man, OMIM®*; Baltimore (MD), 2020;
2. Lek, M.; Karczewski, K.J.; Minikel, E. v.; Samocha, K.E.; Banks, E.; Fennell, T.; O'Donnell-Luria, A.H.; Ware, J.S.; Hill, A.J.; Cummings, B.B.; et al. Analysis of protein-coding genetic variation in 60,706 humans. *Nature* **2016**, *536*, 285–291, doi:10.1038/nature19057.
3. Human Protein Atlas Assays & Annotation Available online: https://www.proteinatlas.org/about/assays+annotation (accessed on Jul 2, 2020).
4. Uhlén, M.; Fagerberg, L.; Hallström, B.M.; Lindskog, C.; Oksvold, P.; Mardinoglu, A.; Sivertsson, Å.; Kampf, C.; Sjöstedt, E.; Asplund, A.; et al. Proteomics. Tissue-based map of the human proteome. *Science* **2015**, *347*, 1260419, doi:10.1126/science.1260419.
5. Thomas, E.; Lewis, A.M.; Yang, Y.; Chanprasert, S.; Potocki, L.; Scott, D.A. Novel Missense Variants in ADAT3 as a Cause of Syndromic Intellectual Disability. *Journal of Pediatric Genetics* **2019**, *8*, 244–251, doi:10.1055/s-0039-1693151.
6. Singh, N.; Gupta, D.K.; Sharma, S.; Sahu, D.K.; Mishra, A.; Yadav, D.K.; Rawat, J.; Singh, A.K. Single-nucleotide and copy-number variance related to severity of hypospadias. *Pediatric Surgery International* **2018**, *34*, 991–1008, doi:10.1007/s00383-018-4330-5.
7. George, M.; Schneuer, F.J.; Jamieson, S.E.; Holland, A.J.A. Genetic and environmental factors in the aetiology of hypospadias. *Pediatric Surgery International* **2015**, *31*, 519–27, doi:10.1007/s00383-015-3686-z.
8. van der Zanden, L.F.M.; van Rooij, I.A.L.M.; Feitz, W.F.J.; Franke, B.; Knoers, N.V.A.M.; Roeleveld, N. Aetiology of hypospadias: a systematic review of genes and environment. *Human Reproduction Update* **2012**, *18*, 260–283, doi:10.1093/humupd/dms002.
9. Qin, X.-Y.; Kojima, Y.; Mizuno, K.; Ueoka, K.; Massart, F.; Spinelli, C.; Zaha, H.; Okura, M.; Yoshinaga, J.; Yonemoto, J.; et al. Association of variants in genes involved in environmental chemical metabolism and risk of cryptorchidism and hypospadias. *Journal of Human Genetics* **2012**, *57*, 434–441, doi:10.1038/jhg.2012.48.
10. Carmichael, S.L.; Ma, C.; Choudhry, S.; Lammer, E.J.; Witte, J.S.; Shaw, G.M. Hypospadias and Genes Related to Genital Tubercle and Early Urethral Development. *Journal of Urology* **2013**, *190*, 1884–1892, doi:10.1016/j.juro.2013.05.061.
11. Fukami, M.; Wada, Y.; Miyabayashi, K.; Nishino, I.; Hasegawa, T.; Nordenskjöld, A.; Camerino, G.; Kretz, C.; Buj-Bello, A.; Laporte, J.; et al. CXorf6 is a causative gene for hypospadias. *Nature Genetics* **2006**, *38*, 1369–1371, doi:10.1038/ng1900.
12. Kurahashi, N.; Sata, F.; Kasai, S.; Shibata, T.; Moriya, K.; Yamada, H.; Kakizaki, H.; Minakami, H.; Nonomura, K.; Kishi, R. Maternal genetic polymorphisms in CYP1A1, GSTM1 and GSTT1 and the risk of hypospadias. *MHR: Basic Science of Reproductive Medicine* **2005**, *11*, 93–98, doi:10.1093/molehr/gah134.
13. Goursaud, C.; Mallet, D.; Janin, A.; Menassa, R.; Tardy-Guidollet, V.; Russo, G.; Lienhardt-Roussie, A.; Lecointre, C.; Plotton, I.; Morel, Y.; et al. Aberrant Splicing Is the Pathogenicity Mechanism of the p.Glu314Lys Variant in CYP11A1 Gene. *Frontiers in Endocrinology* **2018**, *9*, doi:10.3389/fendo.2018.00491.
14. Rubtsov, P.; Karmanov, M.; Sverdlova, P.; Spirin, P.; Tiulpakov, A. A novel homozygous mutation in CYP11A1 gene is associated with late-onset adrenal insufficiency and hypospadias in a 46,XY patient. *The Journal of Clinical Endocrinology and Metabolism* **2009**, *94*, 936–9, doi:10.1210/jc.2008-1118.
15. Lara-Velazquez, M.; Perdomo-Pantoja, A.; Blackburn, P.R.; Gass, J.M.; Caulfield, T.R.; Atwal, P.S. A novel splice site variant in CYP11A1 in trans with the p.E314K variant in a male patient with congenital adrenal insufficiency. *Molecular Genetics & Genomic Medicine* **2017**, *5*, 781–787, doi:10.1002/mgg3.322.
16. Joodi, M.; Amerizadeh, F.; Hassanian, S.M.; Erfani, M.; Ghayour-Mobarhan, M.; Ferns, G.A.; Khazaei, M.; Avan, A. The genetic factors contributing to hypospadias and their clinical utility in its diagnosis. *Journal of Cellular Physiology* **2019**, *234*, 5519–5523, doi:10.1002/jcp.27350.
17. Andresen, J.H.; Aftimos, S.; Doherty, E.; Love, D.R.; Battin, M. 13q33.2 deletion: a rare cause of ambiguous genitalia in a male newborn with growth restriction. *Acta paediatrica (Oslo, Norway : 1992)* **2010**, *99*, 784–786, doi:10.1111/j.1651-2227.2010.01683.x.

18. Garcia, N.M.; Allgood, J.; Santos, L.J.; Lonergan, D.; Batanian, J.R.; Henkemeyer, M.; Bartsch, O.; Schultz, R.A.; Zinn, A.R.; Baker, L.A. Deletion Mapping of Critical Region for Hypospadias, Penoscrotal Transposition and Imperforate Anus on Human Chromosome 13. *Journal of Pediatric Urology* **2006**, *2*, 233–242, doi:10.1016/j.jpurol.2006.03.006.
19. Walczak-Sztulpa, J.; Wisniewska, M.; Latos-Bielenska, A.; Linné, M.; Kelbova, C.; Belitz, B.; Pfeiffer, L.; Kalscheuer, V.; Erdogan, F.; Kuss, A.W.; et al. Chromosome deletions in 13q33–34: Report of four patients and review of the literature. *American Journal of Medical Genetics Part A* **2008**, *146A*, 337–342, doi:10.1002/ajmg.a.32127.
20. Camats, N.; Flück, C.E.; Audí, L. Oligogenic Origin of Differences of Sex Development in Humans. *International Journal of Molecular Sciences* **2020**, *21*, doi:10.3390/ijms21051809.
21. Geller, F.; Feenstra, B.; Carstensen, L.; Pers, T.H.; van Rooij, I.A.L.M.; Körberg, I.B.; Choudhry, S.; Karjalainen, J.M.; Schnack, T.H.; Hollegaard, M. v; et al. Genome-wide association analyses identify variants in developmental genes associated with hypospadias. *Nature Genetics* **2014**, *46*, 957–963, doi:10.1038/ng.3063.
22. Zhang, W.; Shi, J.; Zhang, C.; Jiang, X.; Wang, J.; Wang, W.; Wang, D.; Ni, J.; Chen, L.; Lu, W.; et al. Identification of gene variants in 130 Han Chinese patients with hypospadias by targeted next-generation sequencing. *Molecular Genetics & Genomic Medicine* **2019**, *7*, e827, doi:10.1002/mgg3.827.
23. van der Zanden, L.F.M.; van Rooij, I.A.L.M.; Feitz, W.F.J.; Knight, J.; Donders, A.R.T.; Renkema, K.Y.; Bongers, E.M.H.F.; Vermeulen, S.H.H.M.; Kiemeney, L.A.L.M.; Veltman, J.A.; et al. Common variants in DGKK are strongly associated with risk of hypospadias. *Nature Genetics* **2011**, *43*, 48–50, doi:10.1038/ng.721.
24. Kojima, Y.; Koguchi, T.; Mizuno, K.; Sato, Y.; Hoshi, S.; Hata, J.; Nishio, H.; Hashimoto, D.; Matsushita, S.; Suzuki, K.; et al. Single Nucleotide Polymorphisms of HAAO and IRX6 Genes as Risk Factors for Hypospadias. *The Journal of Urology* **2019**, *201*, 386–392, doi:10.1016/j.juro.2018.07.050.
25. Carmichael, S.L.; Witte, J.S.; Ma, C.; Lammer, E.J.; Shaw, G.M. Hypospadias and variants in genes related to sex hormone biosynthesis and metabolism. *Andrology* **2014**, *2*, 130–137, doi:10.1111/j.2047-2927.2013.00165.x.
26. Vezzoli, V.; Duminuco, P.; Vottero, A.; Kleinau, G.; Schülein, R.; Minari, R.; Bassi, I.; Bernasconi, S.; Persani, L.; Bonomi, M. A new variant in signal peptide of the human luteinizing hormone receptor (LHCGR) affects receptor biogenesis causing leydig cell hypoplasia. *Human Molecular Genetics* **2015**, *24*, 6003–12, doi:10.1093/hmg/ddv313.
27. Rossetti, L.Z.; Glinton, K.; Yuan, B.; Liu, P.; Pillai, N.; Mizerik, E.; Magoulas, P.; Rosenfeld, J.A.; Karaviti, L.; Sutton, V.R.; et al. Review of the phenotypic spectrum associated with haploinsufficiency of MYRF. *American Journal of Medical Genetics* **2019**, *179*, 1376–1382, doi:10.1002/ajmg.a.61182.
28. Hughes, J.J.; Alkhunaizi, E.; Kruszka, P.; Pyle, L.C.; Grange, D.K.; Berger, S.I.; Payne, K.K.; Masser-Frye, D.; Hu, T.; Christie, M.R.; et al. Loss-of-Function Variants in PPP1R12A: From Isolated Sex Reversal to Holoprosencephaly Spectrum and Urogenital Malformations. *American Journal of Human Genetics* **2020**, *106*, 121–128, doi:10.1016/j.ajhg.2019.12.004.
29. White, J.; O'Neill, M.; Sheth, K.; Lamb*, D. Murine RBFOX-2 Haploinsufficiency Parallels Congenital Anomalies in Human Patients with RBFOX-2 Copy Number Variants. *The Journal of Urology* **2020**, *203*, e978, doi:10.1097/JU.0000000000000940.11.
30. Sreenivasan, R.; Gordon, C.T.; Benko, S.; de Iongh, R.; Bagheri-Fam, S.; Lyonnet, S.; Harley, V. Altered SOX9 genital tubercle enhancer region in hypospadias. *The Journal of Steroid Biochemistry and Molecular Biology* **2017**, *170*, 28–38, doi:10.1016/j.jsbmb.2016.10.009.
31. Han, X.-R.; Wen, X.; Wang, S.; Hong, X.-W.; Fan, S.-H.; Zhuang, J.; Wang, Y.-J.; Zhang, Z.-F.; Li, M.-Q.; Hu, B.; et al. Associations of TGFBR1 and TGFBR2 gene polymorphisms with the risk of hypospadias: a case-control study in a Chinese population. *Bioscience Reports* **2017**, *37*, doi:10.1042/BSR20170713.
32. Tannour-Louet, M.; Han, S.; Louet, J.-F.; Zhang, B.; Romero, K.; Addai, J.; Sahin, A.; Cheung, S.W.; Lamb, D.J. Increased gene copy number of VAMP7 disrupts human male urogenital development through altered estrogen action. *Nature Medicine* **2014**, *20*, 715–24, doi:10.1038/nm.3580.

33. Marrocco, G.; Grammatico, P.; Vallasciani, S.; Gulia, C.; Zangari, A.; Marrocco, F.; Bateni, Z.H.; Porrello, A.; Piergentili, R. Environmental, parental and gestational factors that influence the occurrence of hypospadias in male patients. *Journal of Pediatric Urology* **2015**, *11*, 12–9, doi:10.1016/j.jpurol.2014.10.003.
34. Köhler, S.; Carmody, L.; Vasilevsky, N.; Jacobsen, J.O.B.; Danis, D.; Gourdine, J.-P.; Gargano, M.; Harris, N.L.; Matentzoglu, N.; McMurry, J.A.; et al. Expansion of the Human Phenotype Ontology (HPO) knowledge base and resources. *Nucleic Acids Research* **2019**, *47*, D1018–D1027, doi:10.1093/nar/gky1105.
35. Miertuš, J.; Maltese, P.E.; Hýblová, M.; Tomková, E.; Ďurovčíková, D.; Rísová, V.; Bertelli, M. Expanding the phenotype of thrombocytopenia absent radius syndrome with hypospadias. *Journal of Biotechnology* **2020**, *311*, 44–48, doi:10.1016/j.jbiotec.2020.02.011.
36. Rafi, S.K.; Butler, M.G. The 15q11.2 BP1-BP2 Microdeletion (Burnside–Butler) Syndrome: In Silico Analyses of the Four Coding Genes Reveal Functional Associations with Neurodevelopmental Disorders. *International Journal of Molecular Sciences* **2020**, *21*, 3296, doi:10.3390/ijms21093296.
